# Delivering embryos following 10 years of cryopreservation, using unpaired freeze/thaw techniques: A case report

**DOI:** 10.5935/1518-0557.20210015

**Published:** 2021

**Authors:** Priscila Melantonio, Jessica Attivo, Alecsandra P. Gomes, Tatiana C.S. Bonetti, Pedro A.A. Monteleone

**Affiliations:** 1 Monteleone Centro de Reprodução Humana, São Paulo, SP, Brazil; 2 Departamento de Ginecologia. Escola Paulista de Medicina, Universidade Federal de São Paulo, São Paulo, SP, Brazil; 3 Disciplina de Ginecologia - Departamento de Obstetrícia e Ginecologia, Faculdade de Medicina da Universidade de São Paulo. São Paulo, SP, Brazil

**Keywords:** *in vitro* fertilization, long-term embryo cryopreservation, slow freezing, vitrification

## Abstract

Although frozen embryo transfer is a widely established route for assisted reproduction, successful frozen embryo transfer using embryos that have undergone long term cryopreservation remains relatively unexplored, and its efficacy remains a matter of some debate. This case report describes two successful frozen embryo transfer conceptions in the same patient, one after 3 months of cryopreservation and the second 10 years after cryopreservation. These embryos were cryopreserved using the slow freezing technique and were thawed using an unpaired technique (ultra-rapid warming) after 10 years of storage.

## INTRODUCTION

Embryo cryopreservation is widely used in assisted reproduction treatments, and following the first successful conception using frozen embryo transfer (FET) in 1984 ^(Zeilmaker *et al*., 1984)^, both embryo freezing and thawing techniques have been modified and improved over time. The slow freezing technique was the first to be developed and is used to store embryos at the cleavage stage. This technique relies on slow controlled cooling, which is facilitated by the equilibration of the embryos in one or more dehydrating solutions and then using a chamber in a programmable freezing machine, which slowly reduces the temperature at a rate of 0.3-1.0˚C/min. Upon reaching temperatures between -40 and -70˚C, the embryos are moved to liquid nitrogen for longer term storage. Embryos are rapidly thawed using a process of rehydration in decreasing concentrations of cryoprotectant at room temperature ^(Edgar & Gook, 2012)^. Although slow freezing enables adequate cellular dehydration while minimizing intracellular ice crystal formation, embryo survival rates and implantation potential after thawing is generally believed to be impaired ^(Van Landuyt *et al*., 2013)^.

Vitrification was later introduced as an alternative and uses ultra-rapid cooling in very small volumes of cryoprotectant solutions to facilitate the solidification of the cell(s) and the extracellular milieu into a glass-like state without the formation of ice crystals. To induce vitrification high initial concentrations of cryoprotectants in a minimum volume (≤1.0 µL) undergo ultra-rapid cooling by direct contact with liquid nitrogen, and ultra-rapid warming at 37˚C is used to thaw these embryos ^(Edgar & Gook, 2012; Rienzi *et al.*, 2017)^. Although slow freezing does produce reliable results, embryo vitrification has proven to be a more effective alternative, not only because it is simple, cheap and fast, but also because it is possible to cryopreserve embryos at the blastocyst stage, which has been linked to higher survival rates and better clinical outcomes. With vitrification there was an increase in the cumulative rate of live births, as it allowed multiple embryo transfers during the same stimulation cycle. Moreover, it is useful for delaying embryo transfer in patients at risk of ovarian hyperstimulation syndrome or changes in endometrial receptivity, making vitrification the key method in modern assisted reproduction treatments ^(Liebermann, 2017)^.

However, there remains a lack of evidence surrounding the impact of cryopreservation time on embryo survival rates and implantation potential, as most studies focus on embryos that have been cryopreserved for a maximums of five years. Some case reports have been published on long-term embryo cryopreservation, many of which have reported successful outcomes using embryos being cryopreserved for 8 to 20 years ^(Go *et al*., 1998; Revel *et al*., 2004; Dowling-Lacey *et al*., 2011; Yuan *et al*., 2019)^. This case report describes a successful live birth after frozen-thawed embryo transfer, in which embryos were cryopreserved by slow freezing and maintained for 10 years in liquid nitrogen. Given the fact that the time frame of these events meant that while the embryos were cryopreserved using the slow freezing technique they were thawed using ultra-rapid warming at 37°C, which enables us to determine the effects of unpaired techniques as well as the likelihood of using older embryos successfully.

## CASE DESCRIPTION

A couple was admitted for an infertility investigation in February 2009 with primary infertility for over one-year. The woman was 29 years old, with regular menstrual cycles (28 days/5 days), normal karyotype (46, XX) and normal hormone levels (FSH 4.9 IU/mL, LH 4.06 IU/mL, estradiol 59.2 pg/ml, prolactin 5.6 ng/ml, TSH 1.5 mUI/L, free T4 1.06 ng/dl, progesterone 0.4 ng/dl, CA 125 82.6 U/mL). The hysterosalpingography showed that both the right and left ovarian tubes were fixed. The husband was a 31 year-old male, who had been submitted to varicocele surgery in 2005 and presented with a normal karyotype (46, XY) and semen analysis (volume 6 mL, sperm concentration of 46.5 million/ml, with 4% demonstrating normal morphology and 61% progressive motility). The infertility factor was determined to be tubal, and *in vitro* fertilization was indicated.

The ovarian stimulation started on March 17, 2009 using 225 IU recombinant FSH (Gonal F^®^, Merck) for the first three days, and then 150 IU for the next six days. On the eighth day of ovarian stimulation, we started her on pituitary blockage using a GnRH antagonist (Cetrotide^®^ 0.25 IU, Merck), which continued for three days. The final oocyte maturation trigger was recombinant hCG (Ovidrel^®^, Merck), which was administered on the tenth day of the ovarian stimulation. Ovum pickup obtained 34 oocytes, 29 metaphase II oocytes (MII), and 5 immature oocytes (1 at metaphase I and 4 in the germinal vesicle stages). The MII oocyte rate was 85.0%, and all MII oocytes were fertilized by ICSI, using ejaculated sperm from her partner. This produced 24 normally- fertilized (2PN) oocytes (fertilization rate: 83.0%), which were cultured under standard conditions until the second day of development (48 hours, Day 2-D2). A total of 18 embryos were cleaved (cleavage rate: 75.0%), and then scored according to the grading system described by ^Veeck (1999)^. These 18 embryos were then cryopreserved at D2 using the slow-freezing technique. The patients presented with ovarian hyperstimulation syndrome (OHSS) and were treated in the intensive care unit.

About three months later, June 06, 2009, the couple returned to the clinic for FET. The patients received endometrium preparation as standard, with estradiol (E2) and progesterone. This included 200 mcg topical estradiol (Estradot^®^, Norvartis) every three days followed by concomitant vaginal administration of 800 mg micronized progesterone (Utrogestan^®^, Besins Healthcare) per day, as routine. After five days of progesterone exposure, FET was performed. For FET, six embryos were thawed using the rapid method with five surviving (83.3% survival rate) the thawing and cultured to the blastocyst stage (Day 5-D5). Blastocysts were classified according to the Gardner method ^(Gardner & Schoolcraft, 1999)^, and a single top quality blastocyst (grade 3AB) was transferred. The other four embryos did not reach sufficient quality for a new cryopreservation. Serum β-hCG levels were measured nine days after blastocyst transfer with a positive outcome and a healthy newborn male was delivered following 38 weeks of pregnancy.

In 2013, about three years after the birth of their first baby, the female partner was diagnosed with breast cancer. Pathological analysis revealed grade III invasive ductal carcinoma. She underwent mastectomy, chemotherapy, and radiotherapy, followed by daily tamoxifen treatment for the next five years. In 2018, the woman was considered cured and in May 2019, about 10 years after her ovarian stimulation and embryo cryopreservation, the couple returned to the clinic for a second round of FET, when the woman was 39 years old.

The second round of FET was scheduled to commence on September 09, 2019, in order to coincide with her natural menstrual cycle. This menstrual cycle was followed by a series of transvaginal ultrasounds. When the endometrium reached 13.9 mm and the patient had a periovulatory follicle, we transferred the embryo. For that, 12 embryos were thawed using the ultra-rapid warming method, and seven embryos survived (58.3% survival rate). These embryos were cultured under standard conditions until the blastocyst stage and classified according to the Gardner method ^(Gardner & Schoolcraft, 1999)^. One blastocyst (grade 1) was transferred on D5 of development and the other six embryos were maintained in culture until day 6, after which four blastocysts (4BB, 5CB, 3AB, 2) were cryopreserved again, using the vitrification technique. Serum β-hCG levels were measured nine days after blastocyst transfer with a positive outcome and a live female newborn was delivered via induced natural labor on June 06, 2020, following 39 weeks of pregnancy.

## DISCUSSION

Improvements in ovarian stimulation protocols and laboratory methodologies have contributed to an increasing number of successfully cryopreserved embryos. However, there is little data available on the safety of prolonged cryopreservation. Although theoretical models speculate that a mammalian embryo may be stored for several thousand years, as suggested by Edwards and Beard, there is still no direct experimental confirmation of this estimate ^(Machtinger *et al*., 2002)^. The largest study ever conducted on this subject, by ^Riggs *et al*. (2010)^ analyzed 11,768 cryopreserved human embryos and concluded that cryopreservation is a safe procedure with no substantial negative effect related to time. However, the mean time of cryopreservation in this study was less than a year, and the outcomes cannot be extrapolated to long-term storage.

Previous studies have reported live births from embryos cryopreserved for 8 ^(Go *et al*., 1998)^, 10 ^(Wilson *et al*., 2006)^, 12 ^(Revel *et al*., 2004; Quintans *et al*., 2012)^, 13 ^(López Teijón *et al*., 2006; Reed *et al*., 2010)^, and even 20 years ^(Dowling-Lacey *et al*., 2011)^. A recent study analyzed live birth rates after long-term cryopreservation (between 12 and 17 years) in 20 patients (128 embryos thawed) whose embryos were preserved using the slow freezing method. This study reported a blastocyst formation rate of 33% and live birth rate of 17% for cleavage embryo transfers and 27% for blastocyst transfers. Of the 115 embryos thawed, they reported a 74% embryo survival rate and 23 patients underwent successful FET, resulting in eight clinical pregnancies ^(Yuan *et al*., 2019)^. This means that there are currently less than 20 cases supporting the success of long-term embryo cryopreservation. However, the number of unsuccessful FETs following long-term storage is unknown.

In this case report the patient underwent two FET cycles using the same cohort of oocytes. The first FET was performed after a short period of cryopreservation (3 months), and the second after long-term storage (10 years after cryopreservation). Thus, we can compare the embryo survival rate from the first (80%) and second (60%) thaws from the same patient. However, it should be noted that due to the extended period between FET cycles, the freeze/thaw techniques were unpaired, the slow-freezing protocol was used for cryopreservation and ultra-rapid warming at 37°C, used for the second round of thawing because of a lack of appropriate media for rapid thawing at our facility. Suggesting that some of the changes may be related to the methods used.

The established protocols for cooling and warming embryos are based on mitigating the two-factors believed to underlay freezing injuries: (i) damage caused by exposure to concentrated solutions and (ii) intracellular ice formation. Both the cooling and warming rates, as well as the concentration of the solutions are critical for maximizing the survival of the embryos as they prevent some of the deleterious consequences of each damaging factor. The cooling and warming rates are critical to avoid osmotic shock ^(Leibo & Sztein, 2019)^ and in our case, the embryos should be warmed using a rapid method. However we were forced to use the ultra-rapid method, which could lead to increased osmotic shock, thus decreasing the survival rates. The time of cryopreservation itself could also have contributed to the lower survival rate in the second FET.

In summary, the embryo survival and success rates after long-term cryopreservation are still under debate, and may vary based on several factors including the pre-freeze embryo stage, morphology, cryopreservation and thawing methods, and storage time. In this case report the interactions between the long-term storage and unpaired freeze/thaw methods (slow-freezing cryopreservation and ultra-rapid warming) could have contributed to decreased survival and slower embryo development in the second FET. Nevertheless, the couple enjoyed a good prognosis at the time of embryo cryopreservation, which allowed two pregnancies after two single blastocyst transfers, even after 10 years of cryopreservation.
